# Loot

**Published:** 1869-10

**Authors:** 


### Loot.

Prof. J. Lewis Smith, of New York, recommends, in primary bronchitis, for an infant one year old, a teaspoonful of the following mixture, every two or four hours:

℞. Spts. ætheris nitrosi, - - - - - - f ʒ j

Syrupi ipecacuanhæ, - - - - - -

Olei ricini, - - - - - - - aa f ʒ ij

Syrupi tolutani, - - - - - - f ʒ vij

Another eligible formula is the following:

℞ Syrupi ipecacuanhæ, - - - - - - f ʒ ij

Potassæ acetatis, - - - - - - gr. xvj — ʒ ss

Aquæ anisi, - - - - - - - f ʒ xiv

M. Dose: One teaspoonful for an infant of six months. If there is decided febrile action, tincture of digitalis, one or two drops, according to the age, may be added to each teaspoonful.—*Medical Bulletin.*

The *rationale* of this is simply elimination by the kidneys. The so-called *expectorants*, when analyzed in effect, will be found to prove such only in so far as they act as diuretics.

***Treatment in Diarrhœa of Infants.***

Dr. Smith (*Med. News and Library*), in his valuable papers on the "Wasting Diseases of Infants and Children," recommends the following prescription, if the bowels are rather loose, with dark, slimy, offensive stools:

℞ Tinct. opii, - - - - - - - *m* viii

Ol. ricini, - - - - - - - - ʒ j

Syrupi zingib, - - - - - -

Mucilag. acaciæ, - - - - - - - aa ʒ j

M. S.—A teaspoonful three times daily. In the screaming fits, accompanied by constipation, this combination of castor-oil with laudanum is very valuable.—*Medical Record.*

This is another case wherein it will be found that to be effectual elimination by diuresis must be secured. See, on another page, observations on the use of fruits in diarrhœa.

In those cases of Headache resulting from gastric difficulty, eight or ten minims of chloroform given internally will materially assist in removing it; or pour a small quantity on some cotton wool contained in a small glass-stoppered bottle, and apply the mouth of it close to the temple, or behind the ear, as near as possible to the seat of pain, for about five minutes. The effect is usually rapid.

A WRITER in the *London Lancet* recommends the following treatment of DISEASED GUMS: The teeth should be washed night and morning with a moderately small and soft brush; after the morning ablution, pour on a second tooth-brush, slightly damped, a little of the following lotion, and apply it to the affected parts: Carbolic acid, one scruple; rectified spirits of wine, two drachms; distilled water, six ounces. — *Medical Bulletin.*

A very efficient wash and gargle in these cases, and in many cases of stomatitis, pharyngitis, aphthar, etc., is afforded by:

| | | |
|---|---|---|
| ℞ | Sodac Biborat., - - - - - - | |
| | Potassæ Chlorat., - - - - - | aa ℈ ij |
| | Acid. Pyrolig., - - - - - - | ℥ ss |
| | Aq., - - - - - - - | ℥ vss |
| | M. ft. gargar, - - - - - - | |

S. Use as a wash.

MR. R. H. ALLNATT, of Eastbourne, whose monthly letters to *The Times*, on meteorological matters, have now become a recognized institution, gives, in his letter on the Meteorology of July, "a curious and suggestive fact" in reference to OZONE. He says that since the establishment, at Eastbourne, of an effective system of arterial drainage, the manifestation of elemental ozone appears to have been elevated, *pari passu*, to the level of the altered sanitary conditions. A few years ago the chemical tests, after long exposure, exhibited, frequently, feeble signs of reaction; but now they show invariable and decided marks of the presence of this allotropic agent. — *Lancet.*

Which suggests a change in the *type* of diseases, as it is conveniently termed. The catarrhal and inflammatory succeed the malarious and septic. At all events, the ozone finds less to neutralize it.

AT the Imperial Society of Surgery, the other day, M. Broca presented a Peruvian skull, found by Squier in an old tomb of the Incas, in the valley of Yucay, near Cuzco, and belonging to a date anterior to the time of Cortez. The interest of the skull to surgeons lay in the fact that it had been trepanned! The perforation was situated on the left side of the frontal bone; and from the appearance of the surrounding osseous tissue, no doubt remained that the operation had been performed on a living person. M. Nelaton estimated that the patient should have survived the trepanning about eight days. — *Id.*

Another evidence that there is nothing new under the sun.

***Rich Beef Tea.***

THE addition of a small tablespoonful of *cream* to a teacupful of beef tea renders it richer and more nourishing.

It was formerly the habit, in directing beef tea, to order all the fat globules to be carefully skimmed from the top, whereby the "Hamlet was left out." Subsequently, the loss was attempted to be made up by administration of Cod Liver Oil. The cream is an improvement.

IN every 100,000 tons of the WATER supplied to London, the solid impurity averages from twenty-eight to forty-two tons; in Edinburgh, from eleven to fourteen tons; Bristol, twenty-eight tons; Manchester, six tons; Dublin, six tons; and Glasgow, only three tons. — *Exchange.*

It is doubtful if any city on the globe has purer water afforded it than this city of Chicago, and the consequence of this improvement on the past is patent to even the least observant in the general increase of health and longevity. Now let combined efforts be put forth to purify the atmosphere, both in and out of the houses.

M. MAILLOT, ex-president of the Conseil du Sante des Armees, counts as a great service rendered to his country his introduction into Algeria, in 1853, of the treatment of DYSENTERY by large doses of bismuth, warmly recommended by M. Monneret. In that year, four pounds of bismuth were forwarded to that country, at his request, for trial, under his observation. The result has been, that it has grown constantly in favor with the regimental surgeons, who have had it included in the troop medicine-chest; and that, in 1868, 920 lbs. weight were required for the treatment of dysenteric affections tn the army of Algiers. — *British Medical Journal.*

Much of the good effect of the introduction of bismuth in the treatment of dysentery, we are fain to believe, is from its replacing the powerful but injurious medication previously in vogue. Astringents, cathartics and mercurial "alteratives" have less relation to the cure of the disease than the too general reliance upon them would seem to involve. The great question even in dysentery is: *What is the matter?*

Carbolic acid is running almost precisely the same race with creosote twenty-five years ago. Dr. McCall Anderson, of Glasgow, is experimenting on "the virtues of certain new (!) remedies" for cutaneous diseases. Prominent among these are coverings of vulcanized India rubber, and India rubber cloth, and the internal use of carbolic acid. Among others, he published the following cases in the Glasgow *Medical Journal:*

Eczema of hands, erythema papulatum, and psoriasis palmaris he treated with *India rubber gloves.* Several cases of psoriasis he treated by carbolic acid. ℞. Acidi Carbol. Cryst. ʒ iss, Glycerini q. s., aq. distil. ℥ vi, M. Sig.—A teaspoonful in a wine-glass of water, three times a day, on an empty stomach.

W. St. John Coleman, in the *London Lancet*, speaks of his success in treating chronic eczema, eczema rubrum eczema facii, and an obstinate case of impetigo larvalis, with carbolic acid. To an eight-ounce phial of glycerine and water he added about ♏xx of pure carbolic acid, and applied it locally night and morning.

Dr. Moritz Kohn, discussing lupus erythematosus, places most dependence on external remedies. The local treatment he recommends consists in removing the accumulated sebaceous secretion by means of applications of oils and various soaps; the use of "spiritus saponatus kalinûs" (sapon. viridis, ℥ iv; sp. vini. rect. ℥ ij; spir. lavand. ʒ ij); liq. potassæ; carbolic acid and the mineral acids; also sulphur (in powder), solid nitrate of silver, chloride of zinc paste, and the emplastrum mercuriale.—*Journal of Cutaneous Medicine.*

Ointment for Hæmorrhoids, by the late Prof. W. R. Fisher:

| | | |
|---|---|---|
| Take of | Sulphate of Morphia, - - - | three grains. |
| | Extract of Stramonium, - - | thirty " |
| | Olive Oil, - - - - - | sixty " |
| | Carbonate of Lead, - - - | sixty " |
| | Lard Cerate, - - - - | three drachms. |

Rub the extract, if not uniformly soft, with a few drops of water; add the powders and olive oil, and rub till perfectly smooth, and then incorporate them with the cerate.—*American Journal of Pharmacy.*

A better plan is to incorporate the active ingredients with cacao butter, and use as suppositious.

### *Cure for Warts.*

Warts may be readily cured by cutting a hole in a piece of sticking plaster, the size of the wart, applying the sticking plaster so that the wart protrudes, and then using —

| | | |
|---|---|---|
| ℞ | Caustic potash, - - - - - - | ℨ ij |
| | Gum arabic, - - - - - - - | ℨ ss |
| | Flour sufficient. - - - - - | |

M. — Make a paste.

S. — To be left on for a few hours. — *Boston Journal of Chemistry.*

Dr. Boulou has devised the following plaster for rheumatic and neuralgic pains:

| | | |
|---|---|---|
| ℞ | Empl. plumbi, - - - - - - | ℥ xvj |
| | Ext. pini sylvestris, - - - - - | |
| | Ext. Belladonna, - - - - - - | aa ℨ jss |

Spread evenly over fine strong linen, so that every square inch should contain two grains of the active ingredient. — *Medical Archives.*

### *Sulphite of Soda in Chronic Cystitis.*

Mr. L. Willcox, late house-surgeon of King's College Hospital, recommends the use of sulphite in those cases of chronic cystitis where the urine decomposes before it is eliminated. He finds that by the employment of sulphite of soda all the putridity disappears, and the urine becomes clear and colorless. — *Canada Medical Journal.*

Dr. J. Waring Curran recommends the iodide of ammonium in diseases of the glanular system. The following are his prescriptions in Goitre:

| | | |
|---|---|---|
| ℞ | Ammonii iodidi, - - - - - - | gr. xv |
| | Spiritus chloroformi, - - - - - | ʒ ij |
| | Aquæ camphoræ, - - - - - - | ad ℥ viij |

M. S. — ℥ j ter in die.

| | | |
|---|---|---|
| ℞ | Ammoni iodidi, - - - - - - | ʒ ij |
| | Glycerine, - - - - - - - | ʒ ij |
| | Adipis benzoat, - - - - - - | ℥ jss |

Ft. — Unguent. — *Journal of Chemistry.*

### *Hypodermic Injection of the Tincture of Calabar Bean in Epilepsy.*

Dr. F. C. Wilson, of Virginia, reports several cases of epilepsy, which he treated by injecting hypodermically ten to twelve minims of the tincture of calabar bean. He alleges that, in patients having a series of epileptic convulsions, recurring at intervals of eight, ten or twenty minutes, the paroxysms at once ceased, and that they soon dropped off into a quiet sleep.

The doctor says that the calabar bean may be combined with strychnine, thus enabling a much larger dose of each to be given than would be safe if given separately.

We think these experiments sufficient to recommend to the profession its further trial in this disease. Will any one give us the result of their experiments with this remedy? — *Richmond and Louisville Medical Journal.*

### *Dr. Brown-Sequard's Usual Prescription in Epilepsy.*

| | | |
|---|---|---|
| ℞ | Potass. iodidi, - - - - - - | ʒj |
| | Potass. bromidi, - - - - - | ℥j |
| | Ammon. bromidi, - - - - - - | ʒiiss |
| | Potass. bicarb, - - - - - - - | ℈ij |
| | Inf. columbæ, - - - - - - | f℥vi |

M. Sig. — A teaspoonful before each meal, and three teaspoonfuls at bed-time, with a little water. The medicine should be pushed until anæsthesia of the fauces is produced, and an acne-like eruption appears on the neck, face, shoulders, etc. Continue treatment for sixteen months after the convulsions have ceased, an occasional purgative being given.

### *Another Formula of the Same.*

| | | |
|---|---|---|
| ℞ | Potass. bromidi, - - - - - | ʒvj |
| | Ammon. bromidi, - - - - - - - | ℈ij |
| | Potass. bicarb., - - - - - | gr. xv |
| | Tr. columbæ, - - - - - - | ℥iss |
| | Aquæ, - - - - - - - - | ℥iij |

M. Sig. — One to two teaspoonfuls three times a day, before meals, in a little water.

N.B. — The above remedies may prove very serviceable in some cases of emphysema, hooping-cough, and nocturnal emissions. — *Physician and Pharmaceutist.*

### *Hypodermic Injection of Morphia in Sun-Stroke.*

Dr. J. H. Hutchinson (*Pennsylvania Hospital Reports*) reports the very great success which followed the hypodermic injection of the sulphate of morphia as a remedy for the excessive jactitation, restlessness, and convulsions which so often attend sun-stroke. It was used as auxiliary to the ice treatment. — *Id.*

### *Goitre — Hypodermic Injection of Iodine.*

The author treats goitre by hypodermic injections of iodine. He began with four or five drops of the tincture, and gradually increased the quantity, repeating the injections every eight days or so. — *Prof. A. Lücke, in Berliner Klin. Wochenschrift.* — *Id.*

***Spasmodic Asthma of Children.***

Bromide of Potassium, in six grain doses in syrup, every two hours, is worth a trial in this affection. — *Dr. W. Begbie, M. Sondahl.*

***Chronic Catarrh.***

Give five minim doses of tincture of aconite every four hours. Opium has a similar action in such cases. — *Dr. S. Wilks, Id.*

***Unguent for Bronchocele.***

Prof. James R. Wood, of New York, extols the following formula as an ointment in bronchocele and other glandular tumors:

| | | |
|---|---|---|
| ℞ | Ung. stramonii, - - - - - | ℥ ij |
| | Ext. conii, - - - - - - - | ʒ ij |
| | Iodid. potassii, - - - - - - - | ʒ ij |
| | Iodini, - - - - - - - | gr. x |

M. — *Detroit Review.*

***Removal of the Odor of Carbolic Acid.***

A correspondent of the *Lancet* says: "The disagreeable odor left by carbolic acid can be readily removed by means of chloride of lime or Condy's fluid." — *Dental Cosmos.*

***Muriate of Ammonia as a Remedy.***

Dr. Cholmeley * * * confirms the observation of Dr. Anstie in a paper in the December number of the *Practitioner*, as to the great efficacy of the muriate of ammonia as a remedy for neuralgia and myalgic pain. But Dr. Cholmeley goes on to say that with regard to a matter on which Dr. Anstie spoke more doubtfully — the efficacy, namely, which certain authors have ascribed to this drug as an emmenagogue — he has formed, from a large experience, a decided opinion in favor of the utility of this medicine. He is convinced that in a very large number of cases of absent or suppressed menstruation, muriate of ammonia acts in a very direct and powerful manner in establishing or restoring the flux. Dr. Cholmeley has now experimented with the muriate, in doses of 10 to 20 grains, in so large a number of hospital and dispensary patients, that he can not suppose there is any room for fallacy in this conclusion.

***On Muriate of Ammonia as a Remedy for some Nervous Affections.***

The *Edinburgh Medical Journal* thus refers to Dr. Anstie's paper:

"Muriate of ammonia is one of those common-place and unattractive substances which we, in this country, are little apt to credit with extensive remedial properties in disease." We quote the first sentence of an eminently suggestive paper by Dr. Anstie (*The Practitioner*, December, 1868), which treats principally of the employment of this remedy for the relief of (1) various kinds of pain, and (2) of certain cases of suspended secretion dependent on nervous exhaustion. Before very briefly describing some of the applications mentioned, we think it right to state that we are by no means prepared to coincide in Dr. Anstie's therapeutics, in so far as this is founded on physiological data. Under the first class the disease termed *myalgia* is said to be specially amenable to treatment by muriate of ammonia. Doses of from ten to twenty grains are recommended, and by their use this disease may be cured as certainlv as ague by quinia. This class also includes various neuralgias proper, such as *migraine* (usually referred to disorders of digestion) and *clavus hystericus;* both of which Dr. Anstie believes to be distinct and primary neuralgias of the fifth cranial nerve. Of all the internal remedies that can be employed in these headaches, none is apparently so beneficial as the muriate of ammonia, its virtue depending on its mildly stimulant properties. It should be given in the same doses as for myalgia. In *intercostal* neuralgia, and especially in that form met with in suckling women or in phthisical patients, this remedy is also of great value, frequently "pain being relieved in half an hour." It mav also be used with advantage in the milder varieties of *sciatica*, which occur in young and debilitated persons; and in that somewhat obscure form of neuralgia termed *hepatic*. Among the therapeutic applications to relieve suspended secretion, Dr. Anstie expresses his conviction that muriate of ammonia "is the most powerful of all functional restoratives" of suspended bile secretion. He especially recommends it in those cases where the disease is produced by severe and exhausting mental excitement; and mentions that he has seen several instances in which two or three doses of twenty grains have caused a marked recommencement of biliary secretion. All these, and various other therapeutical indications, are founded on the supposition that this remedy is a "pure tonic stimulant" to sensitive and to vaso-motor nerves.

***Lime-Water in the Treatment of Bright's Disease.***

Kuchenmeister recommends, in the treatment of Bright's disease, and of nephritis, after scarlatina, the use of large doses of lime-water, theoretically from its having the property of dissolving proteine. Lyon Médicale details the treatment, and says that caustic lime in solution, or any of the soluble salts of lime, will answer equally well. He has seen the urine increase from

30 grammes to 120 the first day, 180 the second, 300 the third, and up to 1,020 the seventh day, under this influence; sometimes a slight hæmorrhage necessitates the disuse of this treatment; but the quantity of albumen in the urine sensibly diminishes. — *Medical Press and Circular.*

***Amblyopia Cured by Hypodermic Injection of Strychnia.***

DR. JOS. TALKO, of Tiflis, reports (*Klin. Monatsblätter f. Augenheilkunde, Mai*) a very interesting case of amblyopia cured entirely and solely by this method. The doses used were $\frac{1}{12}$ raised gradually to $\frac{1}{4}$ of a grain of nitrate of strychnia; the injection was made in the neighborhood of the affected eye; it seemed to answer best when done in the supra-orbital region. The cure may be said to have occupied about seven weeks, and was then complete. It is remarkable that such large doses, repeated as often as once a week, produced neither local inconvenience nor constitutional poisoning, with the exception of the trivial symptoms. — *The Practitioner.*

***Calabar Bean in Tetanus and Trismus Neonatorum.***

DR. MONTI has been trying this treatment in four cases of tetanus in new-born children, and one case of traumatic origin in a boy four years old. The traumatic case and two of the infants recovered; the remedy was mostly administered by subcutaneous injection. The author remarks on the extreme rarity of favorable results in this disease, and concludes, from his own experience, and that recorded by other observers, that calabar bean offers a much better prospect of positive therapeutic results than does any other drug. The proper dose for subcutaneous injection of an infant a few days old seems to be $\frac{1}{30}$ to $\frac{1}{6}$ grain of the extract; in the boy of four years old, Monti injected as much as $\frac{1}{2}$ grain, and a total of $6\frac{1}{2}$ grains of the extract were given altogether, by stomach and by skin. — *The Practitioner, August*, 1869, *from Jahrb. f. Kinderheilkunde, Mai.*

***Preservation of Dead Bodies.***

AT the meeting of the Imperial Academy of Medicine (June 8), M. Devergie stated that, at the School of Practical Anatomy, bodies injected with a mixture of three parts of glycerine, and one of carbolic acid, had been preserved for several months without giving off any unpleasant odor.

***A New Styptic Collodion.***

M. CARLO PARESI gives, in the *Gazette de Turin*, the following recipe: Collodion 100 parts, carbolic acid 10 parts, tannin 5 parts, benzoic acid 3 parts.

Agitate until a perfect solution is formed. It is of a brownish color, gives a pellicle similar to ordinary collodion, and instantly coagulates blood. — *Indian Medical Gazette.*

Dr. Willard Parker, of New York, is responsible for the following prescription:

| | | |
|---|---|---|
| ℞ | Magnesia carb., - - - - - - | ℥ i |
| | Cret. preparat., - - - - - - | ℥ ss |
| | Ferri carb., - - - - - - | ʒ ij |
| | Sodii chlorid., - - - - - - | ʒ ss |
| | Sodæ carb., - - - - - - | ℥ iij |

M. S. — Two grains and a half at a dose.

It is proper to add that the patient was directed to resume teetotal habits, which had been temporarily laid aside.

The *Investigator*, of this city, thinks the damp weather of the early summer attributable to the eclipse, and the increased prevalence of cholera morbus due to the damp weather, and that Arsen. "stopped the whole train of symptoms short off." In one case Verat. was needed to stop the cramps. We are delighted to read also that, "In this disease, this year, our experience and observation have led us to look upon Arsen. as meeting the *genius epidemicus.*"

It is asserted that acute *Orchitis* may be subdued, in twelve hours, by applying a strong solution of Nitrate of Silver over the entire surface of the swollen part. Meanwhile, it is suggested as desirable to bring the patient under profound sedative influence. In the olden time orchitis was commonly treated by antimonial emetics carried to the production of uttermost nausea. Either plan seems better than the modern heroic one of puncturing the tumor at once.

***Sulphophenic Acid.***

Mr. Joseph Hirsh, in the *Pharmacist*, claims for Sulpho-carbolic acid a disinfecting power over simple Carbolic acid in the proportions of 375 : 225. Aside from its greater efficacy, he says, the point of economy is in favor of Sulpho-carbolic acid, as it can be produced at less cost. This is a point of considerable importance, as the demand keeps Carbolic acid at too high a price for that profuse use needed to secure its satisfactory action as a general disinfectant. For the latter purpose it is said (Squibb) that the impure acid is superior.

"The Sulpho-carbolic acid was prepared by digestion of its two constituents for thirty-six hours. The mixture gradually grew warm, the rise of temperature continuing for twenty-four hours, when it slowly subsided. The mixture of the pure acids assumed, slowly, a greenish color, which finally grew very dark, so as to be easily mistaken for black, as is the case with the aniline black, which, in reality, is a dark green. The crude Carbolic acid grew warm much sooner with the Sulphuric than the pure, due to the decomposition of the impurities by the Sulphuric acid, its color being jet black. It emitted, continually, pungent fumes of Sulphuro-carbolic acid, which were highly volatile and penetrating. Its disinfecting power was tested with putrid blood, and was found to stand between that of Carbolic and Nitrophenic acid. * * * * * Experiments, to determine the comparative disinfecting power of Carbolic and Sulpho-carbolic acid, disclosed the fact that Carbolic acid (the impure used by the city, and, according to Squibb, much superior as a disinfectant to the pure acid) was capable of disinfecting eight thousand parts of Chicago River water completely; the water in greater quantity evolving "strong" volumes of the fetid, sulphureted and phosphorated hydrogen. This last odor adhered to the vessel after emptying the partly disinfected water. The Sulpho-carbolic acid, also crude in the dilution of 1-16000, completely disinfected the same water, taken at the same time from the same vessel, while a further dilution to 1-32000 made the odor of the water appear, but very faintly, while at the same time that of the disinfectant was still plainly perceptible. After emptying the mixture, and filling the vessel, in which the disinfection had taken place, twice with pure water, washing it each time, there still remained a strong odor — but not unpleasant — of the disinfectant, which thus seemed to adhere, with extraordinary tenacity, to the vessel. Disinfection is due to the chemical combination of the disinfectant with the noxious substances, which thus become neutralized, amounting to the same as annihilation, since they then lose their characteristic activity just in the same manner in which this takes place with other chemical compounds in case of neutralization. As a process of chemical combination, disinfection is subject to the usual mathematical rules governing all combinations, and laid down in the tables of equivalents.

"According to these, it is not surprising that the mineral acid compounds of Carbolic acid possess a higher saturating, and therefore disinfecting, power than the Carbolic acid itself.

"Sulpho-carbolic acid may be represented by the formula:

$HO+(C_{12}H_5O,SO_3)SO_3$; *i. e.*, it is made up of two atoms of Sulphuric acid, of which fifty parts, by weight, will neutralize, or in our case, disinfect, as much as 125 parts of Carbolic acid, which is also contained in the above." — *Pharmacist.*

IN order to obtain the 315 grammes of nitrogenous matter which man requires daily, it would be necessary, according to Payen, to consume sixteen dozen oysters, if they should form the sole nutriment. — *Journ. de Pharm. et Chem. Id.*

DR. A. FRŒHDE established the identity of Hydrocarotine and Cholesterine, of which the former seems to be a hydrate. This partly illustrates the nutritive value of carrots. — *Archiv. der Pharmacie. Id.*

EXTRACT of Calabar bean, suspended in gelatine, forms convenient elastic sheets for application to the eye. The dose contained in a small piece should be 0.010 gr. of the bean, or 0.0005 gr. of the extract. — *Arch. der Pharm. Id.*

PROF. BUCHNER, in Munich, demonstrated lately again the transformation of Arsenic into Sulphide of that metal in cadavers of those poisoned with the acid. — *Arch. der Pharm. Id.*

***On the Use of Starchy Food for Infants.***

AT a meeting of the Obstetrical Society of London, July 7. (*Medical Times and Gazette*,) a paper was read, by Dr. Selby Norton, on TEETHING.

In this paper the author advocated the opinion that the maladies usually attributed to teething are due to the wide-spread and unphysiological practice of feeding infants on starch foods. He showed that starch was non-digestible by the infant stomach, partly because no minute division of the starch granules could be effected in the infant's mouth, and partly because, from the mode of feeding, the greater part, at all events, of the starch is passed at once into the infant's stomach without being rendered soluble by the ptyalin of the saliva. The diseases usually ascribed to teething — diarrhœa, convulsions and bronchitis — in the author's experience, never occurred in a naturally fed child; and, on the other hand, they occurred sometimes in the first month, where the teeth obviously could exercise no baneful influence, and they occurred, too, when the gums were quite cool and natural. After considering these diseases at some length, and showing how often they could be directly traced to the irritation of bowel produced by starch food, he concluded by condemning altogether farinaceous food for infants, and advocating the sole use of cow's milk diluted with water.

Dr. T. Ballard said he was pleased to see some one come forward to support the "heretical" doctrine that teething was not a cause of infants' disease—a doctrine he had advocated many years ago. While so far, however, agreeing with the paper just read, he could not coincide in his view that starch was such a patent cause of disorder. He did not think starch, *per se*, was harmful, though of course it was not a substance on which an infant could be reared. With respect to the general subject of infant mortality, he thought that practical good would result from the inquiry if the Society could agree upon some formula of dietary for general recommendation of a simple and intelligible character. He would also lay much stress not only upon the importance of sufficient food, but on the importance of not allowing the bowels to act more than twice in the twenty-four hours. This could be effected by attention to the mode of giving the food; by not allowing an infant to suck without obtaining the food it craves, or to suck too hard to obtain it. In either case the bowels became disturbed and diarrhœa was the result. Should this occur while the child is at the breast, the too frequent motions indicate the necessity of some supplementary feeding; or, if the infant be fed entirely from the bottle, there is probably some defect in its construction or action. Where maternal milk, in sufficient quantity, could be obtained, of course no other food was requisite. Next to this came the milk of some other animal, and, where circumstances required it, to this might be added some preparation of wheaten flour.

Dr. Phillips considered it injudicious to give any farinaceous food to an infant under six months old. The practice was as physiologically incorrect as it was practically found to be hurtful. The paper read had not convinced him that no evils were ever caused by teething; but he quite believed that the evil effects ascribed to teething were often caused or increased by improper feeling. At the Children's Hospital, instructions "How to bring up Babies," had been distributed with the best effect.

Dr. Brunton said that he also objected *in toto* to giving a child farinaceous food up to six or eight months. Up to that age, where suckling could not be carried out, he gave cow's milk and water, sweetened, increasing the proportion of milk as the child grew older.

Dr. Routh said that on no point was there more evidence than against the use of starch for infants before they had teeth. For—1. The assimilation of starch depended on its conversion into sugar by the saliva, but infants secreted no saliva for the first two or three months; 2. In infants dying after the use of starchy food, examination showed that it passed through the alimentary canal unchanged; 3. The alimentary canal of a baby was that of a carnivorous animal; 4. The food supplied to purely herbivorous animals recently born was animal. *Ergo*, starchy food should not be given to infants until, at all events, the appearance of teeth. He could not agree with the recommendation of cow's milk diluted with water, as a good food for infants. The milk, before it was purchased, was generally watered, deficient in cream, acid, and wanting in sugar of milk. If used at all, it must be mixed with lime-water, and sugar of milk added in proportion of half to one ounce of lime-water, and a spoonful of sugar of milk to every half-pint of milk, with one-third water. It should be begun early, even from birth, in all cases where it was clear beforehand that the mother could not nurse long. The idea that it was wrong to mix two milks was fallacious, and his experience had proved to him that the earlier it was begun the more readily the child's stomach bore it, and in nine cases out of ten a child so prepared could be weaned readily and with safety. To one other point only would he refer — the congregation of infants in nurseries. This was a most dangerous practice. The atmosphere generated under these conditions was most baneful, probably from the qnantity of ammonia generated from the urine, as well as sulphuretted hydrogen and other noxious gases from the stools. Children required air, and pure air especially. Their respiration was more rapid than adults. Such congregation of infants was always, therefore, a great cause of infant mortality. Malignant thrush, *muguet*, and contagious diseases spread like fire in such atmosphere. — *Phila. Reporter.*

### *Antiseptic Treatment of Wounds.*

Letter from Dr. J. C. Warren. — *Mr. Editor:* Having recently had the privilege of visiting the wards of Prof. Lister, at Glasgow, it may prove of some interest to the readers of the *Medical Journal* to learn the latest modifications he has made in the antiseptic treatment of wounds.

This subject still continues to excite considerable interest in most English cities, and has been taken up and employed successfully in some of the continental schools. Although this system has been condemned by many distinguished surgeons, it has not been by any means universally so, and still claims several enthusiastic supporters in Great Britain.

It may be as well to touch upon his germ theory of putrefaction and the process of healing by scabbing, although the subject has been very clearly and elaborately exposed by him in a series of articles which have appeared during the last eighteen months in the *Lancet and British Medical Journal.* The germ theory may be briefly stated thus: — suppuration in wounds is caused by an irritation produced by the presence of germs or organisms which find their way into a wound, and there multiply and cause putrefaction.

Putrefaction, then, is the exciting cause of suppuration in a wound: can this be prevented, the largest wound may heal without any secretion of pus. This process of healing, such as may take place in a large lacerated wound of the leg accompanying compound fracture, is not considered by him to be healing by "first intention," nor indeed "by granulation." It is rather an intermediate process. Given a wound sufficiently large and accompanied with sufficient loss of substance to be incapable of healing by first intention — the extravasated blood and serum cover the surface of the exposed parts and form a clot which serves the purpose of a protecting scab. Provided now that no living germ is introduced beneath or penetrates this covering, the cell formation takes place quietly in the parts below, while the clot itself becomes organized in the same manner as a thrombus in an artery. The clot or scab establishes in this way a vascular connection with the parts beneath. Meanwhile, cicatrization continues, and as the edges of the wound approximate each other the scab is compressed on all sides, and finally atrophies and comes away. If it is cut into, however, before union is complete, it will *bleed*. When a wound heals in this manner, no pus whatever is found upon the dressings. They may be stained by the escape of a small amount of serum and what is called a mucous discharge. This fluid, examined carefully under the microscope, is found to contain no pus corpuscles whatever.

The antiseptic treatment of wounds has undergone a variety of modifications since Mr. Lister first began his experiments, some two years ago. Most of these have been described at length in the English journals, and will hardly need repetition here, especially as his present method differs from them in several essential particulars.

The dressings are now changed daily, and the tin plate and the paste have been discarded, and a very thin piece of oil silk and a lac plaster* are used in their place. After an operation the wound is washed with a solution of Carbolic acid, one part to twenty of water, and the edges are brought together by antiseptic sutures. The nozzle of a syringe is then introduced into one end of the wound, which is freely syringed out with the same solution. A strip of very thin oil silk, rendered antiseptic by being dipped into the acid solution, is then placed upon the wound of a size just sufficient to cover it. The object of this is to protect the wound from the Carbolic acid contained in the dressing next

* The receipt, as given us by the New Apothecaries' Company, at Glasgow, is the following:

Take of Shellac 3 parts,
Carbolic acid 1 part.

Dissolve with gentle heat, and spread with machine; when spread, coat with a solution of gutta percha, 1 + 16 of bisulphate carbon.

to come. This consists of the lac plaster. Before application the plaster is stripped off from its cambric, by moistening the cloth in water. This is done in order that the plaster may more easily adapt itself to the parts about the wound. The gutta-percha layer must also be rubbed off. The size of the plaster thus applied is sufficient to overlap the wound an inch or two in all directions. Above this is applied another much larger piece of the plaster, with its cambric on, and the whole is secured by a bandage.

The object which he tries to accomplish is to *blockade* the wound in all directions by dressings exhaling Carbolic acid vapor, while the wound itself is not touched by the acid at all. The small amount of the acid left in the wound soon ceases to exert any irritating influence, and the wound is exposed only to the vapor of the acid which penetrates the oil-silk covering. Mr. Lister has found by experiment that the vapor of the acid which passes through a piece of oil-silk is sufficient to disinfect any animal matter which may be on the other side. Any secretions which exude from the wound and become exposed to the air are thus thoroughly disinfected before they have a chance to regurgitate. The same fate awaits any germ which tries to find its way in with them.

If there is any discharge from the wound the dressings should be changed daily. The upper dressing being removed, the lower layer of plaster, which adheres closely to the skin, is carefully peeled off from one end and with it the oil-silk. As the wound is exposed it is syringed with the $\frac{1}{40}$ solution, and this is continued until the new dressing is applied.

Plaster dressings can not be applied in all cases; for instance, on wounds about the genital organs. In such cases a piece of lint soaked in a solution of one part to five of oil is used, but this must be changed frequently.

We should not omit to add that the parts to be operated upon should be well washed with a weak solution of the acid, and if there are any folds of skin or parts covered with hair in the neighborhood, these should be rubbed hard with the $\frac{1}{5}$ oily solution, to destroy any organisms that may be lurking about.

A word here about the antiseptic ligature and suture. A detailed account of the ligature of arteries on the antiseptic system has been given by Mr. Lister in an article in the *Lancet* of April 3, 1869, and he still continues to employ ligatures prepared in this way. This, in brief, consists in the employment of fine catgut ligatures steeped in an oily solution of the acid of the strength of one part to five, with a small quantity of water diffused through it. It has been found, by experiment, that such a ligature not only does not exert any irritating influence on the parts about, but eventually becomes organized and intimately connected with the outer coat of the artery and the surrounding tissue. At present, torsion is used almost universally in England for all the smaller arteries, and the writer had the opportunity of witnessing an amputation of the breast by Mr. Lister, where not a single ligature was used.

Up to the present time he has contented himself with employing silk for sutures — with one exception, however. This was in an operation for the removal of a small tumor on the forehead, where catgut sutures were used. The wound was dressed antiseptically, and the patient left for the country and was not seen for several days. On his return, the dressings were removed, and the wound was found to have healed without suppuration. The sutures remained, to all appearance, unchanged; but on seizing one of them with the forceps in order to cut them with the scissors, the external portion came away easily, leaving *no trace behind* of the part which had been buried in the edges of the wound. The same was the case with all the other sutures. He is of the opinion that in this case the deep portion of the suture had either become organized or was absorbed. He purposes to experiment further in this direction to see if this result is constant.

The antiseptic dressing has been found to be most successful in the treatment of abscesses, compound fractures, excisions of the breast, and in those wounds to which the dressing can be easily and accurately applied. He has not had uniform success in the treatment of amputations, though he has found them, on the whole, to do much better than when dressed according to old rules. During the last two years that he has employed this system, he has had but one case of erysipelas and two of pyæmia. This, in wards which contain on an average some sixty patients, and in an infirmary which has for its site an old cholera burying-ground, is certainly something to boast of.

The writer has had the opportunity of conversing at length with Mr. Lister on this system, and also with many prominent English surgeons, and can truly say that no where has he seen the details so carefully attended to as in Mr. Lister's wards. Most surgeons, in England, at least, have contented themselves with following his directions in a general way, frequently omitting some important particular. For instance, one writer states that he took great pains to wash out his sponges in water both before and during the operation! The very thing he should have taken care not to do, unless the water had been previously rendered antiseptic.

Whatever the merits of the antiseptic system may be, it is very evident that a proper appreciation of them can never be arrived at without that scrupulous attention to detail which has so frequently been insisted upon by its originator.

Very truly yours, J. C. Warren, M.D.

***Colotomy for Relief of Cancer of the Rectum with Stricture.***

On the 24th of last February, I was called to see Mrs. M., a Scotch lady, aged sixty-four years; mother of eleven children. She had been treated by several surgeons for "inward piles." An examination per rectum revealed a stricture one inch from the anal orifice. She said, "before this bowel trouble, she had never required medical aid; never had any of the troubles of women in child-birth; had the constitution of a *Heeland poney;* took forty-five pills now to open her bowels, of which one several years preceding was sufficient; for several days and nights was constantly on the stool; the discharges were as pipe-stems, mixed with blood and very offensive; with intolerable scalding, burning pain; was frequently two or three weeks without a discharge from bowels; did not think she could survive another day, from the pain and distension; had suffered more or less sixteen years," etc.

In the presence of the Drs. Boerstler, I dilated the stricture (with an instrument similar to the dilator used in glove fingers), and during the next twenty-four hours she passed five chamber-pots full of fæces; passed corn eaten in August; and fell thirty-six inches in circumference. This afforded relief, but the hæmorrhagé was nearly five pints, and precluded a repetition of future dilatation.

This operation, however, afforded an opportunity for diagnosis, and that was cancer of the rectum, the stricture a part of that disease—prognosis death. The treatment, concentrated food, and to relieve pain Svapnia (Dr. Biglow's purified opium).

From February to July her condition, from proper diet and anodynes, was tolerably comfortable, with only occasional paroxysms of pain and great tenderness. So painful was the simple touching of the stricture, as that she positively refused any future examinations, alleging a continuance of the pain following the touch of the fingers for days. In July, with the excessive heat, pain, tenderness and anorexia, we hoped the case would terminate, and as she always said, "she was not afeard til' dee;" we gave the Svapnia in doses as high as fifty grains in twenty-four hours, and even these doses failed of relief. I then proposed *Colotomy as a palliative measure;* and as the thermometer then was in the nineties, we awaited a change in the temperature, until the morning of the day of the eclipse, 7th of August last, hoping, as had frequently before resulted, that we should have cooler weather. In the presence of Drs. Boerstler, Wagonbuds, Kinsman, Carpenter and others, the patient being fully anæsthetized, I performed Amussat's operation. I think it unnecessary to detail the various steps of my operation, but refer the reader to Erichsen's Surgery, or for greater satisfaction to Prof. Blackman's detail of two cases, in *Cincinnati Journal of Medicine*, February and March, 1866. I followed Amussat as closely as possible, and found just the place "where the difficulties of the operation commence," and where Prof. Blackman truly says, "here you will meet trouble." I met it by the protrusion of more than a pound of fat; I removed it; then in the triangular space, in front of the quadratus lumborum found the colon; brought it to the surface by two ligatures passed through it, used them with other two in fixing it to the skin, having previously made a slit of one inch and a half in the bowel, and dressed the wound as directed. I made an incision of nine inches through the integuments, thus giving ample room; and by the use of Carbolic acid, not one drop of matter ever formed, and I did not remove the sutures until the tenth day. (Commend me to Carbolic acid as a dressing.) On the seventh day, fæces were discharged from this artificial anus, and two passages a day with immense flatulence (however gradually lessening), have continued and now exist.

I will state one fact, that probably aided me and is not recorded. I used a hair pillow under the abdomen, the patient lying on her face. This protruded the flank and threw the colon outward. Could I have done so, I would have injected into the colon a quart or so of gruel, but the stricture precluded it. The hair pillow was the only substitute and answered a good purpose. I found a large impaction of magnesia in the colon, and on tracing the rectum down encountered the cancerous nodules.

This is now the sixth week of the operation, and in my long professional life, no operation or duty performed, gives me more pleasure. There is perfect rest, return of appetite, with Svapnia gradually reduced from fifty grains a day to four, and only some trouble to keep open the orifice as it is closing with cancer cells. For this purpose I use a silver dilator and compressed sponge, and should my patient not live one month more, I am assured it will be the most comfortable one for years.

I prefer the space that might be occupied by details of the operation, to be used in presenting arguments in favor of or against the operation of Colotomy. It is a formidable one, and has friends and decided opponents. It may in some unfortunate case decide the surgeon to perform Colotomy, and would be more profitable to him than my detail of one case, which detail he will find as referred, better than I can describe it, but the arguments may not be so convenient. I am indebted to the research of Prof. Blackman for my extracts, and will use them liberally.

Prof. Gross, third edition, Comprehensive System of Surgery, 1864, vol. 2., p. 619, says: "It ought to be discarded as among the obsolete devices of surgery." "I have performed the operation but twice, and I do not think any thing could even induce me to attempt it again," and offers an apology for even describing it. In another place he calls it "a criminal procedure." Mr. Pirrie, of Aberdeen, in his last edition of Surgery, quotes Prof. Gross, with approbation.

Prof. Henry H. Smith, in his great work on Principles and Practice of Surgery, vol. 2, p. 441, says: "At first sight it would appear that this operation must evidently expose the patient to loss of life, and can but prolong it only at the expense of a most loathsome disease. That the bowel may be thus opened, is certainly an evidence of the efforts of the surgeon towards relieving the defect of nature; but the results are by no means positive that the operation can assure to the patient, even a continuance of a miserable existence."

I might refer to many other leading authorities, who, if they do not denounce the operation as "criminal," offer no arguments in its favor, but prefer a quotation from Mr. Teele, of Leeds, who, in his valuable article published in Costillos' Cyclopædia of Practical Surgery, vol. 2. p. 219, lays down the following doctrine: "It has been maintained that the surgeon is not justifiable in prolonging the life of an infant, in whom the anus and rectum are imperforate, upon the loathsome condition of an artificial anus; but such an opinion cannot be justified upon any principle of morality, since an imperative obligation rests upon the surgeon to employ to the utmost of his ability, the means placed at his command for the relief of human suffering and the prolongation of life. In obstructions from carcinoma, the operation has been objected to, on the ground that the disease itself must in a short time prove fatal; but if the obligation of the surgeon to prolong life in infancy be imperative, surely he will feel it no less binding in reference to adult age, when he considers how vast and important may be the concerns which hang upon the prolongation of life in an adult human being for a few months, or even for a few days or hours."

Now let us try Prof. Gross' sweeping denunciations of this operation by his instructions for the performance of amputations in *malignant diseases*. See his remarks under the head of Operative Surgery and the Qualifications of a Surgeon, p. 473, vol. 1, op. cit: "It is a sad and humiliating spectacle to see a surgeon cut off a limb, or remove a cancerous tumor, merely for the sake of having it said that he performed an operation. I am daily shocked by the reports of cases of the extirpation of malignant growths in the hospital, as well as in the private practice of this and other countries. The question may well be asked, when will such silly and unmeaning, or to use the proper expression, criminal proceedings cease to disgrace our profession, and to shock our sensibilities?" Now, should I prove Prof. Gross himself guilty of such "criminal proceedings," my reader may be shocked. We quote from page 496, vol. 1, where he speaks of amputations in general. "To cut off an arm at the shoulder, on account of an incipient cancerous affection of the head of the humerus, when the elbow, forearm, hand and fingers are all perfectly natural and glowing with health, unconscious, so to speak, of the fate that awaits them, is enough to sicken the stoutest heart, and to discourage the boldest operator. If there is a more disagreeable task than this, I am ignorant of it, *and yet I would not shrink from its performance* (the italics are our own) *even when there is but a faint prospect of prolonging life, if only for a few months.*"

He also, at page 501, repeats the same doctrine, and at page 477 recommends the amputation of a deformed foot, as he "can see no difference between the physical suffering that is induced by a diseased bone, and the mental distress that results from a distorted foot." In varicocele he recommends the operation, because "its mental effects destroy the happiness of the patient," and this operation, in his own skillful hands, has proved fatal. Again, at page 924, vol. 2, he advises operative interference in cases of malignant disease of the breast, when, "as not unfrequently happens, the patient's mind, filled with gloomy forebodings, is in a state of constant depression and despondency. Here the knife acts as a cordial; hope is revived; new life springs up, and for a while, at least, there is relief from suffering." In many other cases he would operate as "a palliative," even to remove the effluvia which poisons the air the patient breathes. We could quote further, but our position is conceded. If any further proof were needed, we would refer to Velpeau's testimony to Prof. Bennett, of Edinburgh, in speaking of operations for relief of *malignant diseases*, when he says that "to my former list I could now add nine hundred and ninety-one cases."

Mr. Erichsen says, in his London edition of his *Science and Art of Surgery*, page 1,029, that he witnessed Amussat's first operation in Paris in 1839, and since that period it has been performed nearly fifty times (forty-seven — mine the forty-eighth case) — and that when the operation has been followed by fatal results, peritonitis does not seem to have exercised any material influence, but death from previous disease, on the constitution or changes from previous disease in the bowel, rather than to the operation itself, "which appears occasionally to have been uselessly done at the last extremity." He says "we should, therefore, have less hesitation in performing the extra-peritoneal (Amussat's operation) in an early stage of those cases in which it is called for, than we should if the section itself were attended with any serious risks to the patient's life."

Mr. Henry Smith, in his *Articles on Diseases of the Rectum*, would not advise the operation "in the last stage of the disease," even though sudden and complete obstruction should occur, but it should take place in the early stages of the disease; and further says, the surgeon is only doing his duty if he recommends a procedure, which, although in itself surrounded with difficulty and danger, *may* yet prolong life for several months, at least, and allow the patient to die, at last, in comparative ease." This is the whole case, and is all the advocates of Colotomy expect.

I could detail the cases of Curling in his invaluable work on the *Treatment of Painful Cancer of the Rectum without Obstruction*, and quote, in conclusion, his words: "The operation is not intended to cure the disease, but for a purpose second in importance only to cure, namely, to relieve immediate suffering, and to prolong life." If the brightest pathologist of his day, Broussais, had lived to the period of the discovery of anæsthetic agents, he might have been made comfortable and his useful life prolonged by Colotomy. He was right in his diagnosis of his own case—"*tout est dans le rectum*"—as, after his death, four inches of the rectum, in its entire circumference, was found diseased, and the abdominal viscera, pectoral organs and brain were perfectly sound.

I close by a quotation from Curling: "The operation of Colotomy may be required not only on account of the difficulty experienced (in cancer) in obtaining relief from the bowels, but also *in consequence of the extreme misery produced by the disease*, and the surgeon is not justified in withholding from his patient even temporary relief from the great miseries I have described, which may be offered by this operation." I could add similar statements from Dr. Walter Hugh Walshe, of University College Hospital, London; Dr. Wm. Brinton, in his Croonian Lectures on Intestinal Obstructions; Mr. Samuel Solly's Surgical Experiences, London, 1865; Dr. Williams, of Dublin; Callisen, (who operated perpendicularly); Dr. N. Ward (who introduces a tube and fills the colon with fluid); and Gunther's *Lehre von dem operationem am Bauche*, with its truthful drawings illustrating the operation, *et mulitos alios*, but my only purpose is to show a firmness in the profession in this, as in many (not many equal it) diseases to give the patient the benefit of an operation, blessed in its ultimate results, however horrible to the eye of some in the act of its performance, or its subsequent annoyances from an artificial anus.

*Lancaster, Ohio.* Dr. Tom O. Edwards.

***Chloral.***

Dr. Liebreich's recent paper, at the Paris Academy of Sciences, on the action of Chloral, says it is a trichloride of aldehyde, and is soluble in water, and as in this solution it exercises no irritant effect, it is very suitable for absorption into the economy. It takes its place between Ether and Chloroform as an anæsthetic.

Experiments made on rabbits have given the most satisfactory results. Profound and calm sleep has been maintained for eight or ten hours, and Dr. Liebreich adds that the rabbits on awakening have manifested none of the usual results, and have proceeded to eat with avidity. — *Dublin Med. Press and Circular.*

### *Bromide of Potassium for the Sleeplessness of Infants.*

M. Moutard-Martin has communicated to the French Academy of Medicine a memoir on "Some Applications of the Bromide of Potassium to the Medicine of Young Infants." Every one, he observes, admits the possession of sedative properties by the bromide, and in this direction it has become one of the most useful substances in the Materia Medica. Bearing in mind its hyposthenic action in erethism of the nervous system, and its innocuity, even in large doses, he believed that it might be employed with advantage in some of the pathological conditions of very young children. Among these, sleeplessness, alike mischievous to the infant and wearying to the nurse, is one of very common occurrence. The child does not seem otherwise ill, but has a very great insufficiency of sleep both by day and night, or only at night. Where a great variety of means has failed to remove this sleeplessness, the bromide succeeds in a remarkable manner, and M. Moutard-Martin adduces in his paper several cases in proof. His conclusions are: 1. The bromide of potassium given in small doses (from five to twenty centigrammes) is very well tolerated by young infants. 2. By its sedative action it cures *insomnia* in these cases. 3. Administered to infants suffering from the accidents of detention, such as restlessness, *insomnia*, cough, etc., it frequently relieves these; and it is probable that its employment, regulated with prudence, would sometimes prevent the occurrence of convulsions. 4. It should not be administered to infants when suffering from diarrhæa. 5. In certain exceptional cases, in which nervous erethism is predominant, its action is prompt and decisive.—*Medical Times and Gazette*, Dec. 12, 1868.

### *Use of Oil of Turpentine in Parasitic Diseases of the Head and in Traumatic Erysipelas.*

For the destruction of the parasites which cause many scalp diseases, Küchenmeister has recommended alcohol, which retards the development of spores and fungi; but several experiments have proved that the operation of alcohol does not extend to the fungi vegetating in the hair-follicles. Tincture of iodine acts more certainly than alcohol on the fungous growths developed in the hair follicles, as in herpes tonsurans, but it is necessary to shave the whole head, and to apply the tincture repeatedly, until the epidermis has been removed three or four times, and even then there are often disappointments in the cure, which in the most favorable cases requires 102 days. According to Professor Von Erlach, painting with oil of turpentine is much more certain and more rapid in its operations in these scalp affections, herpes tonsurans being cured within fifty days, and several cases of mentagra being cured in the same manner within a week. Since according to recent researches traumatic erysipelas is to be reckoned among the infectious diseases, and often resists the ordinary methods of treatment, Professor Lūcke endeavors to destroy the infectious matter by a drug which penetrates deeply into the tissues, and for this purpose he paints the affected parts with oil of turpentine. He records a case in which this plan was successfully tried, and he states that it was also successful in eight other cases, the erysipelas disappearing in two or three days, whereas, formerly he had observed the most dangerous symptoms to occur in similar attacks. A very striking circumstance was the rapid fall of the temperature, in connection with the disappearance of the symptoms, and this reduction of the temperature was always manifested on the first application, and was the more certain in proportion to the energetic use of the remedy, and, therefore, it was especially observable after rubbing.—Prof. VON ERLACH AND LUCKE, Berne.—*American Journal of the Medical Sciences.*

### *M. Latour on Animal Vaccination and Vaccinal Syphilis.*

M. LATOUR, in the *Union Médicale*, after adverting to the isolated position in which this long debate has left M. Depaul, lays down the following propositions as the legitimate results that flow from it: 1. The degeneration of the Jennerian virus is far from beiug proved. 2. There does not exist a single authentic example of vaccinal syphilis, properly so called. 3. The excessively rare cases of syphilis inoculated by vaccination are explicable by conditions which completely exonerate the vaccine virus from all injurious mixture. 4. A large number of pretended examples of syphilis following vaccination justify the most serious doubts as to the accuracy of the diagnosis. 5. Animal vaccination, simply as another source of lymph, is deserving of encouragement, although it possesses no real or sensible advantage over vaccination from arm to arm.—*London Medical Times and Gazette.*

